# Supplementary material for: Machine Learning-driven Fragment-based Discovery of CIB1-directed Anti-Tumor Agents by FRASE-bot
Source: Res Sq. 2023 Aug 16:rs.3.rs-3197490. Preprint. [Version 1] doi: 10.21203/rs.3.rs-3197490/v1 (PMC10462244; doi:10.21203/rs.3.rs-3197490/v1)
Supplement: Supplement 1 [file NIHPPrs3197490v1-supplement-1.pdf]

Supplementary Table S1. Bit strings for 20 amino acids used in residue triplet screening.

Residue name	Code	Positive ionisable	Negative ionisable	H-bond acceptor	H-bond donor	Aromatic	Hydrophobic	Volume
Alanine	ALA	0	0	0	0	0	1 0 0	1 0 0
Arginine	ARG	0	1	0	0	0	0 0 0	1 1 1
Asparagine	ASN	0	0	1	1	0	0 0 0	1 1 0
Aspartic Acid	ASP	1	0	0	0	0	0 0 0	1 1 0
Cysteine	CYS	0	0	1	1	0	1 1 0	1 1 0
Glutamic Acid	GLU	1	0	0	0	0	0 0 0	1 1 1
Glutamine	GLN	0	0	1	1	0	0 0 0	1 1 0

Glycine	GLY	0	0	0	0	0	0 0 0	0 0 0
Histidine	HIS	0	1	0	1	1	0 0 0	1 1 0
Isoleucine	ILE	0	0	0	0	0	1 1 0	1 1 0
Leucine	LEU	0	0	0	0	0	1 1 0	1 1 0
Lysine	LYS	0	1	0	0	0	0 0 0	1 1 1
Methionine	MET	0	0	0	0	0	1 1 1	1 1 1
Phenylalanine	PHE	0	0	0	0	1	1 1 1	1 1 1
Proline	PRO	0	0	0	0	0	1 0 0	1 0 0
Serine	SER	0	0	1	1	0	0 0 0	1 1 0
Threonine	THR	0	0	1	1	0	1 1 0	1 1 0
Tryptophan	TRP	0	0	0	1	1	1 1 0	1 1 1
Tyrosine	TYR	0	0	1	1	0	1 1 0	1 1 1
Valine	VAL	0	0	0	0	0	1 1 0	1 1 0
